# Interface strain in vertically stacked two-dimensional heterostructured carbon-MoS_2_ nanosheets controls electrochemical reactivity

**DOI:** 10.1038/ncomms11796

**Published:** 2016-06-03

**Authors:** Landon Oakes, Rachel Carter, Trevor Hanken, Adam P. Cohn, Keith Share, Benjamin Schmidt, Cary L. Pint

**Affiliations:** 1 Department of Mechanical Engineering, Vanderbilt University, Nashville, Tennessee 37235, USA; 2 Interdisciplinary Materials Science Program, Vanderbilt University, Nashville, Tennessee 37235, USA; 3 Vanderbilt Institute of Nanoscale Science and Engineering, Vanderbilt University, Nashville, Tennessee 37235, USA

## Abstract

Two-dimensional (2D) materials offer numerous advantages for electrochemical energy storage and conversion due to fast charge transfer kinetics, highly accessible surface area, and tunable electronic and optical properties. Stacking of 2D materials generates heterogeneous interfaces that can modify native chemical and physical material properties. Here, we demonstrate that local strain at a carbon-MoS_2_ interface in a vertically stacked 2D material directs the pathway for chemical storage in MoS_2_ on lithium metal insertion. With average measured MoS_2_ strain of ∼0.1% due to lattice mismatch between the carbon and MoS_2_ layers, lithium insertion is facilitated by an energy-efficient cation-exchange transformation. This is compared with low-voltage lithium intercalation for unstrained MoS_2_. This observation implies that mechanical properties of interfaces in heterogeneous 2D materials can be leveraged to direct energetics of chemical processes relevant to a wide range of applications such as electrochemical energy storage and conversion, catalysis and sensing.

## Abstract (web summary)

Two-dimensional materials are promising for electrochemical energy storage, conversion, catalysis, and sensing. Here the authors leverage strain engineering using a two-dimensional stacked carbon-MoS_2_ material to control chemical storage pathways in MoS_2_ upon lithium metal insertion.

## Graphical abstract

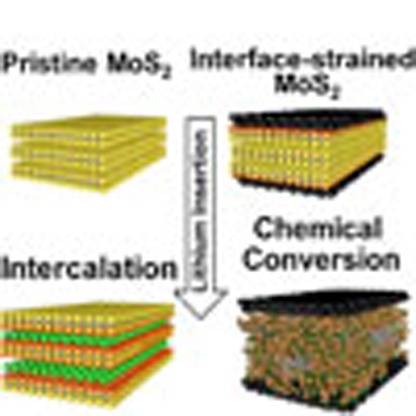

2D graphene and transition metal dichalcogenide (TMDC) materials have captivated researchers in the past decade owing to a set of unique physical and chemical properties that deviate from their bulk analogues and the exploitation of these properties in broad applications^1^. Specifically for electronics, vertical integration of 2D materials enables logic component design with high on-off ratio and promise for three-dimensional (3D) electronics that progress beyond silicon^2^,^3^,^4^,^5^,^6^,^7^,^8^. In semiconductor electronics, engineering 2D TMDC materials using strain applied at an interface has been shown to strongly modulate the bandgap and band structure, which results in modified electrical and optical properties for strained materials^9^,^10^,^11^,^12^,^13^,^14^. For monolayer MoS_2_, the bandgap is observed to shift by up to 15 meV under tensile strain of up to 4.8% (ref. 15), and for multilayered WSe_2_ the bandgap transitions from indirect to direct under strain of up to 2% (ref. 16). Theoretical efforts have further emphasized strain-enabled broadband absorption and photodetection in MoS_2_, even though this has not yet been experimentally realized^17^. The intersection of strain-engineered properties of 2D materials and heterostructured vertically integrated interfaces of 2D materials presents an engaging research area for next-generation electronics. Nonetheless, such materials and research directions remain elusive for many other applications of 2D materials.

On this front, 2D materials and specifically 2D TMDCs have demonstrated excellent performance in a range of electrochemical applications including lithium- and sodium-ion batteries^18^,^19^,^20^, photocatalytic conversion^21^,^22^,^23^ and biosensing applications^24^,^25^,^26^. Unlike electronic devices, these applications require bulk-like quantities of 2D materials—a challenge aided by recent developments in the liquid exfoliation and assembly of layered TMDCs^27^,^28^,^29^. However, addressing how properties in heterostructured or complex 2D materials can impact chemical processes responsible for electrochemical applications is hampered by uniform material fabrication routes that can be used on scales required for electrochemical measurements. As a result, the impact of strained interfaces in 2D materials on chemical and electrochemical processes remains virtually unstudied. Only recently has an observation emerged that compressive strain on Pt catalysts can improve the oxygen reduction reaction capability of Pt relevant to fuel cells^30^. In this manner, 2D materials provide an ideal test bed for the understanding of how interfaces and strain can impact electrochemical processes, motivated by pioneering efforts in the field of semiconductor electronics.

This is the focus of this report, which documents that interface strain measured over statistical quantities of stacked 2D carbon-MoS_2_ heterostructured nanosheets can be directly correlated with distinct differences in chemical processes occurring in 2D materials. In particular, combining optical and electrochemical techniques we demonstrate that strain engineering of interfaces can enable control of the energetic pathway for the chemical conversion of MoS_2_ into electrochemically active Mo and Li_2_S_*n*_ species during reaction with lithium. This highlights the important role that mechanical strain can have in engineering energy storage processes in materials.

## Results

### Fabrication of interface-strained materials

**Figure 1:**
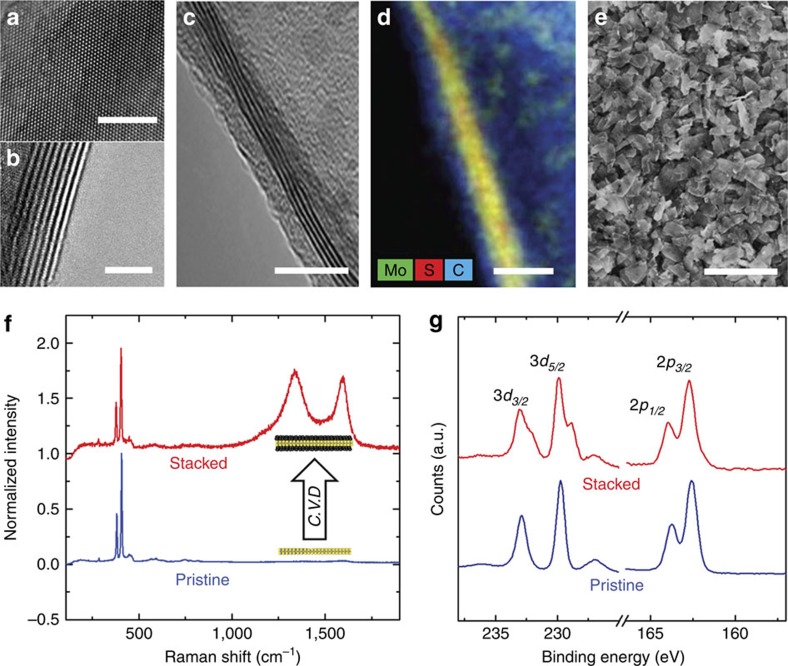
Synthesis of vertically stacked carbon-MoS_2_ heterostructured nanosheets. (**a**,**b**) TEM images of pristine exfoliated MoS_2_ nanosheets. (**c**) Edge-view TEM image of a vertically stacked carbon-MoS_2_ (C-MoS_2_) nanosheet. (**d**) Energy-dispersive X-ray spectroscopy analysis of a stacked C-MoS_2_ nanosheet. The coloured inset describes the colour assignment of each mapped element, overlapping Mo and S signals appear yellow in the image. (**e**) Scanning electron microscopy image of an electrophoretically assembled electrode material formed with stacked C-MoS_2_ nanosheets. Scale bars, 4 nm (**a**,**b**) 10 nm (**c**,**d**) and 1 μm (**e**). (**f**) Raman spectroscopy and (**g**) X-ray photoelectron spectroscopy of pristine MoS_2_ nanosheets and stacked C-MoS_2_ nanosheets.

MoS_2_ nanosheets were produced through liquid exfoliation of bulk MoS_2_ powders in *n*-methyl-2-pyrrolidone (NMP) solvents and subsequent centrifugation. Transmission electron microscopy (TEM) of a representative exfoliated MoS_2_ nanosheet is shown in Fig. 1a,b. Interlayer spacing of ∼0.61 nm is observed for MoS_2_ nanosheets, with thicknesses ranging from 2–15 atomic layers (Supplementary Fig. 1, Supplementary Note 1). Ultrathin carbon layers are grown directly on the MoS_2_ surface through MoS_2_ catalysed decomposition of C_2_H_2_ precursors using a temperature ramp chemical vapour deposition process (Supplementary Fig. 2) that is capable of gram-scale batch processing. This generates vertically stacked architectures where ultrathin carbon layers are formed on both sides of the MoS_2_ nanosheets, with a representative TEM image of this architecture shown in Fig. 1c, and corresponding elemental analysis map in Fig. 1d. To produce an appreciable mass of material for electrochemical tests, electrophoretic deposition was used to assemble the vertically stacked carbon-interfaced MoS_2_ (C-MoS_2_) nanosheets into conformal films on metal substrates from NMP dispersions following chemical vapour deposition (Fig. 1e; Supplementary Fig. 3, Supplementary Note 2). All aspects of synthesis and processing of C-MoS_2_ materials are chosen to be compatible with scalable processing, and ongoing research efforts to improve the simplicity and scalability of liquid exfoliation can further improve this^31^. Raman spectroscopy analysis (Fig. 1f) of vertically stacked C-MoS_2_ nanosheets indicates that vertical stacks maintain an identical signature of crystalline MoS_2_ following the carbon synthesis based on *E*_2g_ and *A*_1g_ modes (300–500 cm^−1^). The carbon layers exhibit a significant amount of *sp*^*3*^ hybridized carbon atoms (∼1,320 cm^−1^, or D-band) relative to *sp*^*2*^ carbon species (∼1,580 cm^−1^, or G-band). Whereas this indicates the presence of carbon, X-ray photoelectron spectroscopy (XPS) indicates the emergence of both a peak in the sulfur *2p*_*3/2*_ and *2p*_*5/2*_ spectra at low-binding energies, as well as shoulders in both Mo *3d*_*5/2*_ and *3d*_*3/2*_ peaks that represent a modulation to the local bonding environment in the crystal (Fig. 1g). Notably, the existence of the low-energy shoulder in the sulfur *2p* and Mo *3d* peaks is consistent with the formation of the metallic 1T-phase of MoS_2_ produced from the strain-induced distortion of sulfur atoms observed previously (Supplementary Fig. 4, Supplementary Note 3)^32^. This analysis collectively supports the formation of a distinct carbon-MoS_2_ stacked interface, where mismatch of the in-plane lattice spacing between MoS_2_ and carbon will induce significant interface strain that propagates into the MoS_2_ nanosheet from the C-MoS_2_ interface.

**Figure 2:**
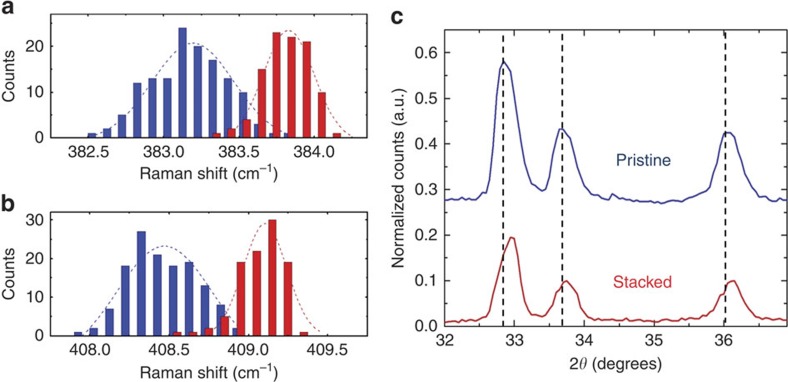
Interface strain in vertically stacked 2D C-MoS_2_ nanosheets. Distributions from Raman spectroscopy maps comprising>100 individual scans showing average shifts in the MoS_2_ (**a**) *E*_2g_ and (**b**) *A*_1g_ modes due to strain induced by a lattice mismatched carbon-MoS_2_ interface. The dotted lines overlaying each distribution represent Gaussian fits to the distribution data. (**c**) X-ray diffraction analysis of vertically stacked C-MoS_2_ nanosheets indicating stacking-induced strain in low-index planes. The following planes can be assigned to the X-ray diffraction spectra: 2*θ*=32.6° is (100), 2*θ*=33.5° is (101) and 2*θ*=35.8° is (102). The vertical dotted lines emphasize the difference in peak positions between the two spectra.

To assess strain in the vertically stacked 2D material, statistical Raman spectroscopy mapping comprising over 200 individual scans in separate areas was performed on the *E*_2g_ and *A*_1g_ modes of MoS_2_, which are highly sensitive to tensile or compressive strain^33^,^34^,^35^. (Fig. 2a,b) On the basis of the peak-to-peak analysis of Raman mode distributions, blue-shifts of ∼0.66 cm^−1^ and ∼0.59 cm^−1^ were observed for the *E*_2g_ and *A*_1g_ modes, respectively. Asymmetry in these modes is expected and attributed to stronger electronic coupling to the *A*_1g_ mode^36^. This yields an ∼0.1–0.2% compressive strain based on relative *A*_1g_ mode shifts in accordance with previous studies^9^,^34^, supporting the presence of interface-induced compressive strain on the MoS_2_ material. This is further confirmed using X-ray diffraction which demonstrates a similar ∼0.1% compressive strain due to vertical stacking based on analysis of the (100) and (110) low-index planes of MoS_2_ (Fig. 2c). We anticipate this measured strain to be present at both the interface and on the interior of the nanosheets as they possess a thickness well below the critical layer thickness at which strain relaxation occurs^37^.

### Controlling electrochemical processes using strain

**Figure 3:**
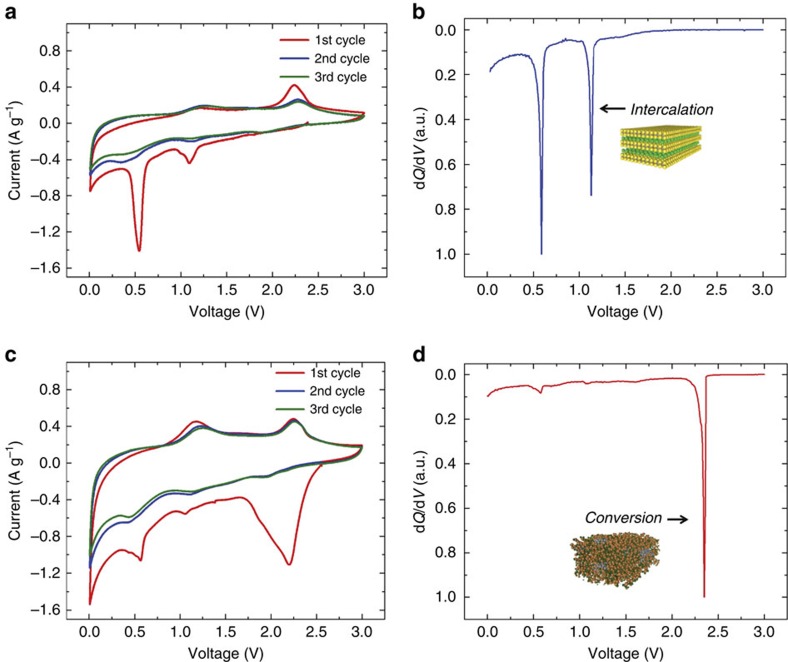
**Electrochemical assessment of strained carbon-MoS**_**2**_ **heterostructured nanosheets.** (**a**) CV and (**b**) normalized differential capacity measurements for MoS_2_ under lithium insertion and extraction, with an arrow indicating the potential of the intercalation reaction that occurs in pristine MoS_2_ materials. (**c**) CV and (**d**) normalized differential capacity plots for vertically stacked C-MoS_2_ nanosheets, with an arrow indicating the electrochemical signature of direct chemical conversion.

To assess how the vertically stacked architecture and interface strain influences electrochemical processes, we combined electrophoretically assembled vertically stacked 2D C-MoS_2_ nanosheets with Li metal electrodes, and a 1.0 M lithium hexafluorophosphate solution in ethylene carbonate and diethyl carbonate electrolyte, and compared the electrochemical properties against similar electrode materials produced with pristine MoS_2_ nanosheets (Supplementary Figs 5,6, Supplementary Notes 4,5). Cyclic voltammetry scans of these electrodes and corresponding differential capacity plots based on galvanostatic measurements are shown in Fig. 3. As these two electrode materials differ only by the presence of a vertically stacked C-MoS_2_ interface, electrochemical data indicates significant changes to the chemical processes occurring on lithium insertion into the MoS_2_ material. For pristine MoS_2_ nanosheets, two subsequent reactions are observed, with the first one at ∼1.1 V and the second at ∼0.55 V versus Li/Li^+^. (Fig. 3a,b) This is consistent with the known pathways for insertion of lithium into pristine MoS_2_, which occurs first through an intercalation reaction (1.1 V) that follows


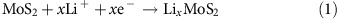


where MoS_2_ undergoes a transition from a semiconducting 2H phase to the metallic 1 T phase due to strain-induced deformation of the crystal from lithium ion insertion. Following this transition, the MoS_2_ can undergo a subsequent conversion reaction at lower voltages (0.55 V) that follows


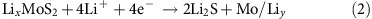


**Figure 4:**
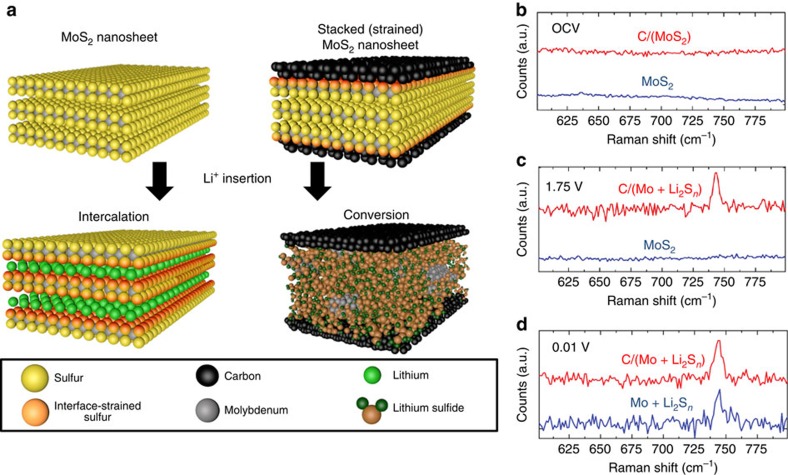
Strain-induced modulation of chemical conversion in vertically stacked C-MoS_2_ nanosheets. (**a**) Scheme showing the different chemical states between interface-strained MoS_2_ nanosheets and pristine MoS_2_ nanosheets after Li insertion, showing chemical conversion for stacked C-MoS_2_ and intercalation for pristine MoS_2_. *Ex situ* Raman spectroscopy confirming the electrochemical signature of chemical conversion based on the Raman mode of polysulfides at 746 cm^−1^ is presented for both materials at (**b**). Open-circuit voltage (OCV), (**c**) 1.75 V and (**d**) 0.01 V versus Li/Li^+^. Notably, at 1.75 V versus Li/Li^+^, the vertically stacked C-MoS_2_ nanosheets have undergone chemical conversion, whereas the pristine MoS_2_ nanosheets remain unconverted.

In contrast to this, insertion of lithium into the vertically stacked C-MoS_2_ nanosheets yields a chemical reaction evident at ∼2.3 V versus Li/Li^+^ based on both CV and diferential capacity curves (Fig. 3c,d) which is close to the open-circuit voltage (OCV) of the device. This reaction proceeds in the absence of Li intercalation into MoS_2_ and no significant signature of lithium insertion at lower voltages occurs such as in pristine MoS_2_ nanosheet electrodes. This highlights the presence of a chemical storage process occurring in vertically stacked C-MoS_2_ materials that is not observed in pristine MoS_2_ materials (Fig. 4a) and that interface-induced strain can directly trigger chemical conversion at practical voltages. It is important to note that these results distinguish the stacked C-MoS_2_ configuration from simple mixtures of carbon nanomaterials and MoS_2_ nanosheets which have been reported previously^38^,^39^,^40^,^41^,^42^, in two key ways, namely that the grown carbon layer induces interface strain not possible in weakly adhered mechanically mixed materials that modulates the chemical reactivity of the MoS_2_ nanosheet toward chemical conversion, and that the stacked configuration provides a physical barrier to inhibit polysulfide dissolution into the electrolyte in a manner consistent with a yolk-shell confinement strategy^43^. However, the benefits reported in other studies by combining MoS_2_ and carbons through mixing are also maintained in our study as evidenced by galvanostatic studies indicating better rate capability and improved storage capacity at high rates for the C-MoS_2_ electrodes (Supplementary Fig. 7). In addition, whereas the correlation between X-ray diffraction, Raman spectroscopy and XPS indicate the presence of strain due to the mismatch of in-plane lattice parameters of the stacked materials below the critical thickness, charge transfer effects between the C and MoS_2_ materials can play a role in the origin of strain measured using these techniques. We also observe the lack of any electrochemical signature of lithium intercalation into the ultrathin carbon coating, implying that stacked C-MoS_2_ nanosheets exhibit differences from pristine nanosheets that can be explained by compressive strain imposed on the 2D MoS_2_ material.

To address the effect of the C-MoS_2_ interface on the electrochemical properties during lithium insertion, Raman spectroscopy of the electrophoretically assembled MoS_2_-based electrodes at different cathodic potentials was carried out (Fig. 4b). This analysis is possible due to the preparation of electrodes in a manner that does not require binder materials often used in conventional battery electrodes that can overwhelm the desired Raman spectroscopic features. At OCV conditions, both electrode materials exhibit only the native Raman modes of MoS_2_. Cathodic scans from OCV conditions to 1.75 V versus Li/Li^+^—an energy below the Li^+^ insertion reaction observed for vertically stacked C-MoS_2_ nanosheets, yields no change for the pristine MoS_2_ nanosheets, but the emergence of a distinct Raman peak at 746 cm^−1^ is observed for the stacked C-MoS_2_ nanosheets. This mode is due to the formation of lithium polysulfides (Li_2_S_*n*_ for 4≤*n*≤8) that can be attributed to chemical conversion of the MoS_2_ into reaction products in a manner consistent with equation (2) (ref. 44). Correspondingly, a decrease of the MoS_2_ *A*_1g_ and *E*_2g_ modes are observed as expected from conversion (Supplementary Fig. 8, Supplementary Note 6). This confirms that the high voltage ∼2.3 V signature in the CV and differential capacity curves for vertically stacked C-MoS_2_ nanosheets (Fig. 3c,d) is a chemical conversion process similar to that which is known to occur at low voltages (0.55 V) in pristine MoS_2_. To further support this, cathodic scans were continued down to 0.01 V versus Li/Li^+^ for the pristine MoS_2_ electrode, where the signature of conversion is evident due to the presence of the Raman mode at 746 cm^−1^. Despite the difference in voltage of the conversion reactions between MoS_2_ and C-MoS_2_, similar Raman spectroscopic signatures of the polysulfide conversion product and similar post-conversion redox energetics in both cases implies that the resulting chemical state of the material in both conversion reactions is the same. To further support this, we performed *in situ* electrochemical impedance spectroscopy (EIS, Supplementary Fig. 9, Supplementary Table 1), where EIS analysis was performed at the same voltages as those assessed using Raman spectroscopy. EIS studies elucidate a picture consistent with Raman spectroscopy results that indicates the electrochemical signature of conversion in different voltage regimes for the pristine MoS_2_ and the stacked C-MoS_2_, respectively (Supplementary Note 7).

## Discussion

On the basis of the combined spectroscopic and electrochemical analysis, a picture emerges emphasizing the role of interface strain on lithium insertion in vertically stacked C-MoS_2_ materials. In the pristine semiconducting 2H phase of MoS_2_, conversion of MoS_2_ nanosheets cannot occur at voltages above where intercalation occurs (1.1 V versus Li/Li^+^) and can only proceed in a two-step chemical process following an intercalation reaction. As many researchers have discussed the promise of MoS_2_ nanosheets for battery cathodes, the requirement of a conversion reaction that is near the reduction potential of lithium is highly impractical in full-cell architectures. In a vertically stacked C-MoS_2_ nanosheet, the interface strain due to lattice mismatch in a carbon-MoS_2_ solid–solid interface leads to an average ∼0.1% compressive strain that propagates into the MoS_2_ nanosheet lattice and enables control of the energetics of chemical conversion. In this regard, interface strain provides the appropriate energetic landscape to sustain direct conversion at voltages that enable pairing with conventional anode materials such as silicon or graphite which give promise to practical incorporation of this device in a full-cell battery configuration. This distinctive difference in the chemical pathways achieved during lithium insertion as a result of interface strain is illustrated in Fig. 4a, where the chemical state of the 2D materials is drastically different following a cathodic scan to 1 V.

The broader use of controlled interface mechanics to modify chemical pathways in low-dimensional materials presents an unexplored horizon for engineered low-dimensional nanostructures. This utilizes 2D materials as a framework to merge concepts of strain engineering with mechanochemistry to control the energetics of chemical processes relevant to energy storage, energy conversion, catalysis, and sensing applications. Building from our results showing that strained interfaces in vertically stacked C-MoS_2_ nanosheets can modulate electrochemical reactivity, we envision a new paradigm for material design that exploits interface strain and mechanics as a versatile toolbox to modulate performance of designer 2D materials in diverse applications.

## Methods

### MoS_2_ nanosheet synthesis

In all, 500 mg of bulk MoS_2_ powder (Aldrich, particle size<2 μm) was added to 50 ml of 1-methyl-2-pyrrolidinone (Aldrich, 99.5% anhydrous). The solution was sonicated in a bath sonicator for 12 h using 8 × 90 min intervals. After sonication, the solution was centrifuged at 2,000 r.p.m. for 40 min. The upper 2/3 of the supernatant was removed and subsequently centrifuged for 1 h at 5,000 r.p.m. before the excess supernatant was removed and the accumulated nanosheets were placed under vacuum overnight to completely evaporate the solvent.

### Vertically stacked carbon-MoS_2_ nanosheet synthesis

MoS_2_ nanosheets contained within an alumina boat were placed into a 1-inch tube furnace and the tube was evacuated to 2 mTorr. A gas mixture of 100 standard cubic centimeters per minute (sccm) Ar and 20 sccm H_2_ maintained at atmospheric pressure were introduced during ramping to a temperature 750 °C. At 750 °C, 2 sccm of acetylene (C_2_H_2_) was introduced for 10 min followed by a temperature ramp to 850 °C and an additional 10 min soak, and a final ramp to 950 °C for 10 min. After this process, the acetylene was turned off and the furnace cooled back down under a flow of argon and hydrogen, where materials were removed.

### Electrophoretic deposition

A total of 20 mg of vertically stacked C-MoS_2_ or pristine MoS_2_ nanosheets were suspended in 20 ml NMP and sonicated for 30 min. Following this, a stainless steel spacer was placed at 0.5 cm from a 1 × 1 cm stainless steel counter electrode. A voltage of 30 V was applied for 30 min before carefully removing the coated steel electrode from solution and placing this into a vacuum chamber overnight to dry the sample.

### Electrochemical device fabrication and testing

Electrochemical half-cell devices were assembled in an argon glovebox using CR 2,032 stainless steel coin cells purchased from MTI. The coated steel discs were separated from a lithium metal anode by a 2,500 Celgard separator saturated with a 1 M LiPF_6_ in 1 g per 1 ml solution of ethylene carbonate and diethyl carbonate. Cyclic voltammetry scans and galvanostatic measurements were performed using a Metrohm autolab multichannel testing system.

### Material characterization

Raman measurements were performed using a Renishaw inVia confocal Raman spectrometer. Micro-Raman maps were collected using a 532 nm laser. Before characterizing the electrodes after electrochemical testing, coin cells were disassembled in an Ar filled glovebox and the electrodes washed with an EC/DEC solution. TEM analysis was performed by drop casting a solution of pristine or stacked MoS_2_ materials onto amorphous carbon TEM grids and then imaged using an FEI Osiris TEM at a beam voltage of 200 kV. X-ray diffraction was performed on powder samples using a Scintag XGEN 4,000 system with a CuKα (wavelength=0.154 nm) radiation source. XPS was collected using a PHI 5,000 Versaprobe. Measurements were collected at a 45° takeoff angle with a 100 μm spot size with charge neutralization.

### Data availability

The data that support the findings of this study are available from the corresponding author upon request.

## Additional information

**How to cite this article:** Oakes, L. *et al*. Interface strain in vertically stacked two-dimensional heterostructured carbon-MoS_2_ nanosheets controls electrochemical reactivity. *Nat. Commun.* 7:11796 doi: 10.1038/ncomms11796 (2016).

## Supplementary Material

Supplementary Information Supplementary Figures 1-9, Supplementary Table 1, Supplementary Notes 1-7 and Supplementary References
