# Supplementary material for: Interface strain in vertically stacked two-dimensional heterostructured carbon-MoS2 nanosheets controls electrochemical reactivity
Source: Nat Commun. 2016 Jun 3;7:11796. doi: 10.1038/ncomms11796 (PMC4895792; doi:10.1038/ncomms11796)
Supplement: Supplementary Information — Supplementary Figures 1-9, Supplementary Table 1, Supplementary Notes 1-7 and Supplementary References [file ncomms11796-s1.pdf]

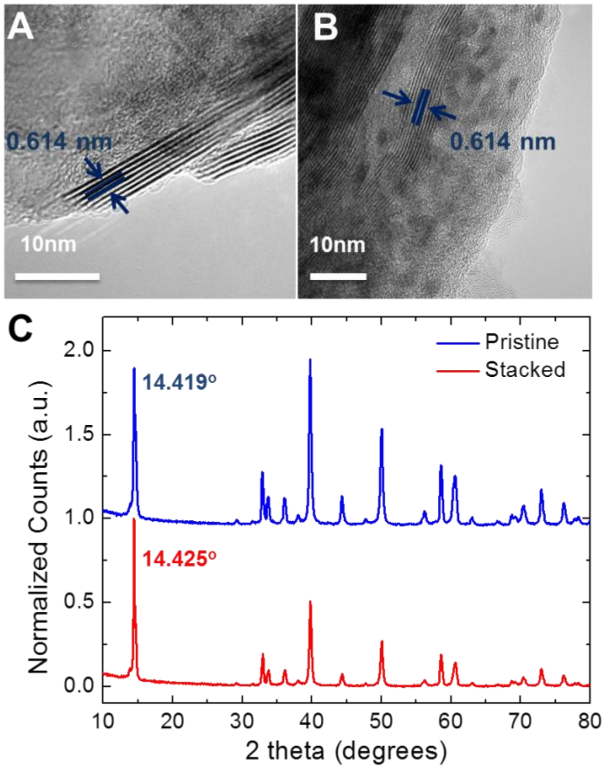

Supplementary Figure 1. MoS_2 structural characterization. Transmission electron microscopy analysis of (a) pristine and (b) carbon-coated MoS_2 nanosheets. Textual insets in the figure depict the interplanar spacing calculated from the X-ray diffraction analysis in (c).

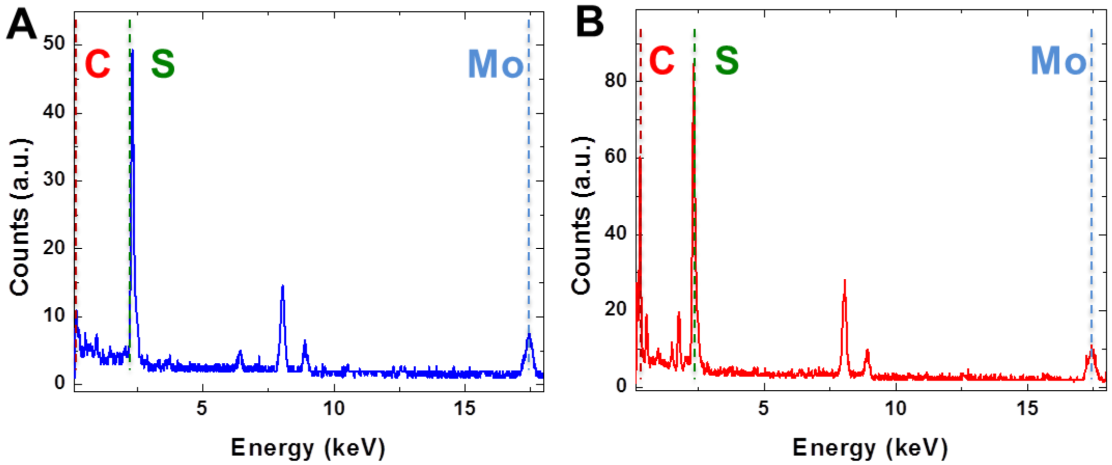

Supplementary Figure 2. Elemental composition. Scanning transmission electron microscopy energy dispersive X-ray spectroscopy analysis of (a) pristine and (b) stacked MoS_2 nanosheets from. The presence of a significant amount of carbon material is observed by the increase in intensity for the carbon k-alpha X-ray at 282 eV in stacked materials.

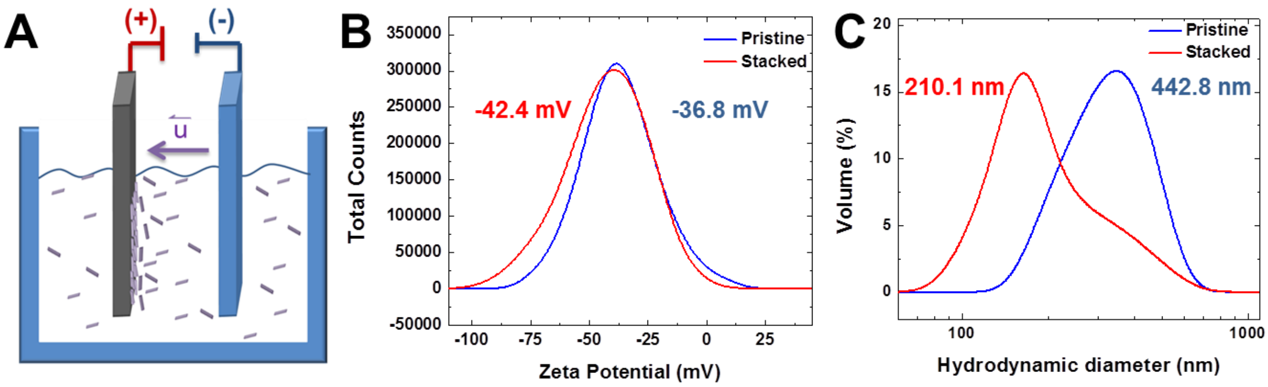

Supplementary Figure 3. Solution properties for stacked and pristine MoS_2 nanomaterials. A schematic illustration of the EPD process is presented in (a). Zeta potential measurements for both materials is presented in (b) along with measurements of the hydrodynamic diameter in (c).

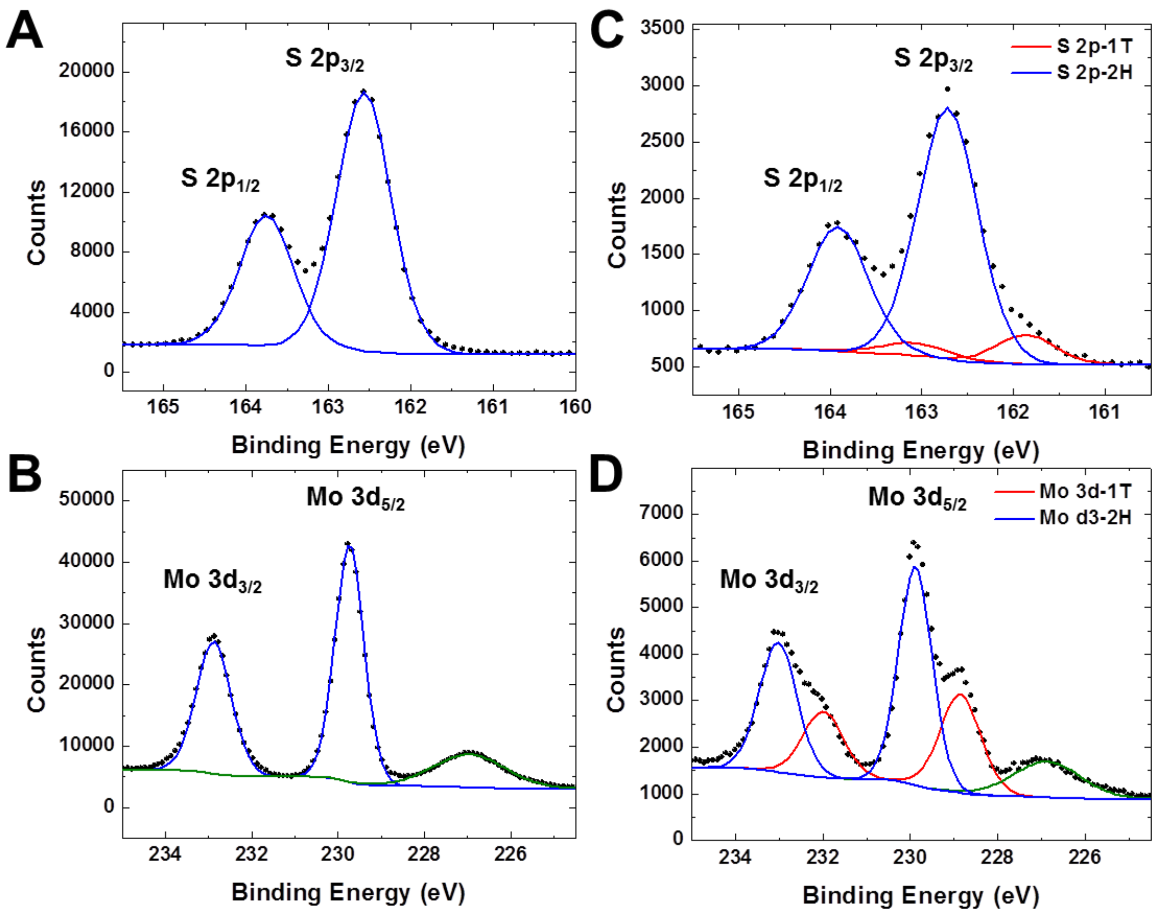

Supplementary Figure 4. **Fits to the XPS data.** The experimental data along with corresponding fits to the XPS data for (a,b) pristine and (c,d) stacked material systems.

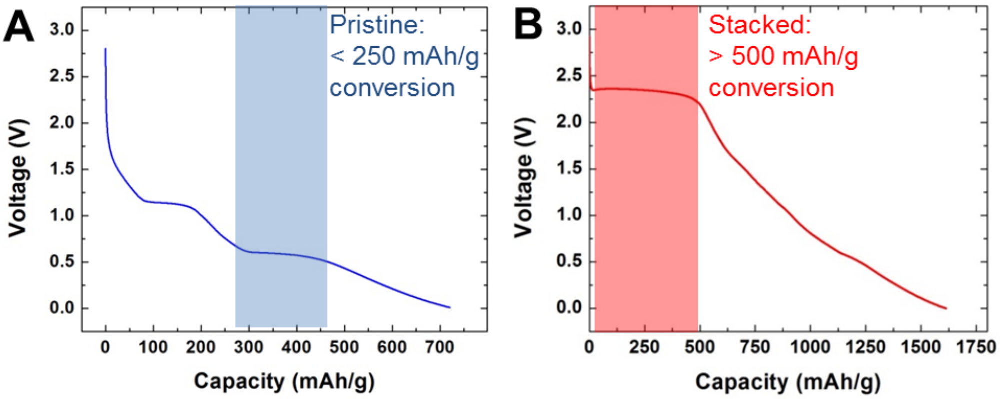

Supplementary Figure 5. **Determination of conversion reaction capacity during lithiation.** Initial discharge curves at a rate of 100 mA/g for (a) pristine and (b) stacked MoS_2 materials. Shaded regions highlight the portion of the discharge curve that corresponds to the conversion of the material to the electrochemically active components.

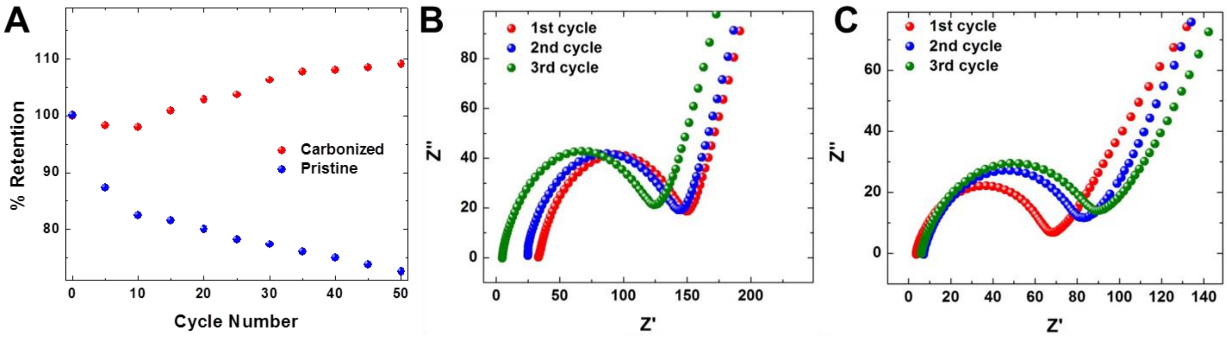

Supplementary Figure 6. Capacity retention during device operation. (a) Cycling performance of the pristine and stacked MoS_2 materials operated at a rate of 100 mA/g. Nyquist plots after the first, second, and third discharge cycles are presented for (b) pristine and (c) carbonized materials.

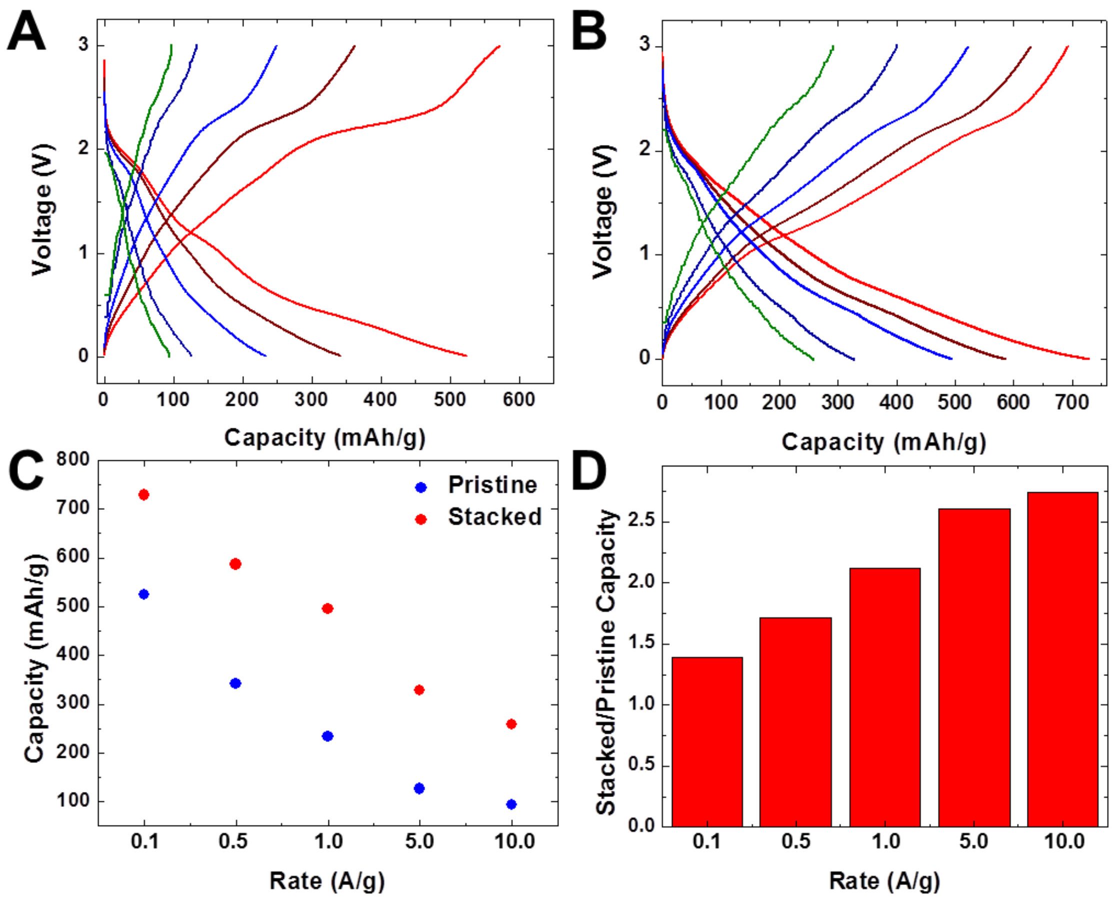

Supplementary Figure 7. **Rate performance.** Charge discharge curves for (a) stacked and (b) pristine structures. A comparison of the capacity at each rate for both materials is provided in (c) along with an analysis of the ratio of the stacked material capacity compared with pristine in (d).

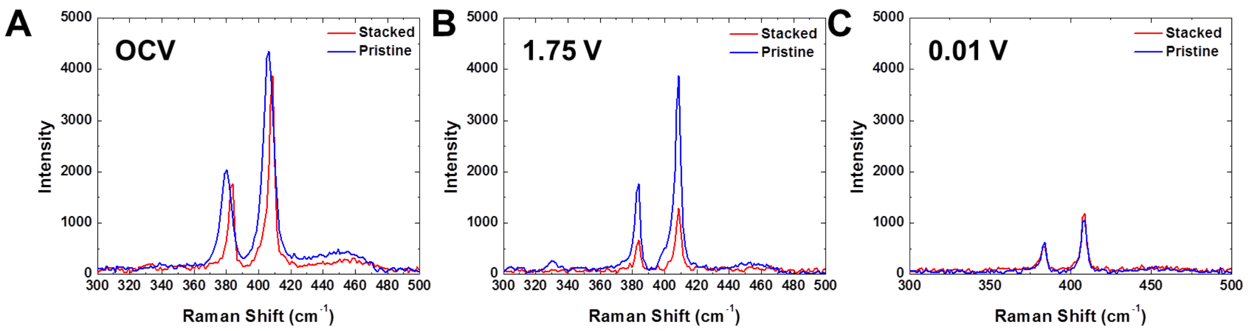

Supplementary Figure 8. MoS_2 Raman intensity during initial discharge. A_{1G} and E_{2G} intensities for stacked and pristine materials (a) at open circuit voltage conditions, (b) after discharge to 1.75 V and the conversion of strained MoS_2 materials, and (c) at 0.01 V after complete conversion in the pristine material system.

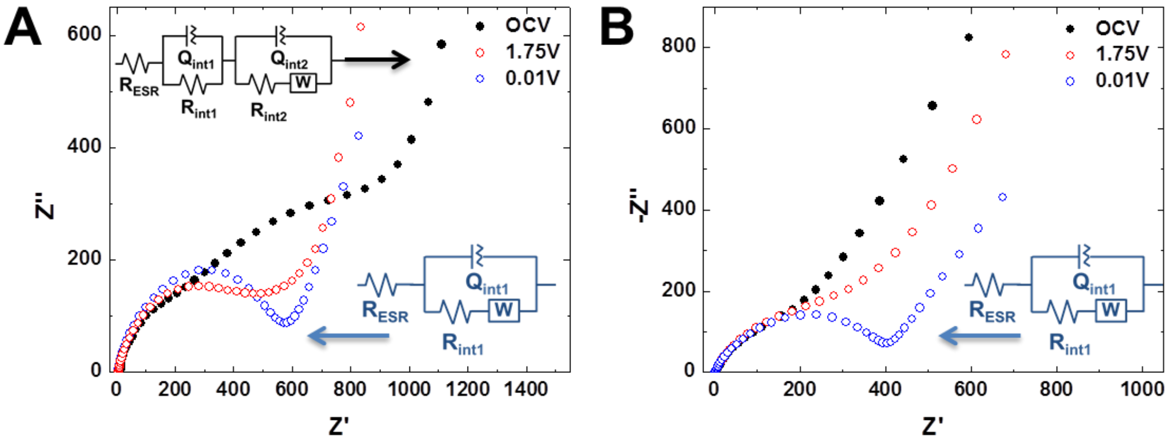

Supplementary Figure 9. **Impedance spectroscopy analysis of the initial conversion reaction .** Nyquist plots obtained from EIS measurements are presented for (a) stacked and (b) pristine materials along with the corresponding circuit provided as an inset.

Supplementary Table 1

	ESR (Ω)	R_{int1} (Ω)	R_{int2} (Ω)
Stacked (OCV)	6.62	450	238
Stacked (1.75 V)	6.58	438	N/A
Pristine (0.01 V)	3.01	385	N/A

Calculated values from equivalent circuit fits shown in Supplementary Figure 9

Supplementary Notes

Supplementary Note 1. Extended Analysis on Strain Measurements of MoS_2

TEM images of pristine MoS_2 NS and vertically stacked MoS_2 NS is presented below in Figure S1a and Figure S1b, respectively, along with corresponding XRD analysis in Figure S1c. The average nanosheet thickness was determined to be 6.5 nm +/- 3.6 nm by averaging measured nanosheet thicknesses across 20 separate TEM images. It is evident that the process of applying the stacked carbon layers presents minimal deformation to the underlying MoS_2 structure as

evidenced by the negligible change in d-spacing between pristine (0.614 nm) and stacked (0.614 nm) MoS_2 layers calculated by the application of Bragg's law to the XRD data for the (002) plane. Previous studies have shown that liquid phase exfoliation results in MoS_2 NS materials with a similar d-spacing[1, 2]. The effect of vertical stacking on the elemental composition of the material was investigated through energy dispersive X-ray (EDS) analysis and the results are presented below in Figure S2a for pristine and Figure S2b for stacked structures. After the stacking process, a large carbon peak appears in the coated samples while the relative amounts of molybdenum and sulfur remain unchanged implying no change to the elemental composition of the underlying MoS_2 layer as a result of the stacking process.

Strain measurements from the XRD data were calculated using an application of Bragg's law to the peak position of the low index peaks:

$$n\lambda = 2d\sin\theta \quad (1)$$

Where n = 1 and λ represents the wavelength of CuK_{α}, approximately 0.154 nm. For the (100) plane, a d-spacing of 0.1420 nm was calculated for the pristine samples compared with a spacing of 0.1418 nm in stacked structures indicating a compressive strain of 0.11%.

Material strain may also be deduced by measuring the phonon energies of a mode m, ω_m, which changes frequency as the lattice expends at a rate determined by the Gruneisen parameter, γ_m:

$$\gamma_m = -\frac{1}{\omega_m}\frac{\partial\omega}{\partial\epsilon} \quad (2)$$

Where the strain $\epsilon = \epsilon_x + \epsilon_y$. Due to the presence of very controlled experimental studies of this system, we have based our strain measurements on the experimental values provided by Hui *et al.*[3] which calculate a shift in wavenumber of 4.65 cm^{-1} per 1% strain in the E_{2g} mode, indicating an average 0.14% strain for our system as calculated by Raman measurements.

Supplementary Note 2. Solution Processing of MoS_2 Suspensions

Fabrication of electrode materials was carried out directly from solution using electrophoretic deposition (EPD) and a schematic of the EPD process is depicted below in Figure S3a. EPD is advantageous to forming electrode materials comprised of nanostructured materials due to its proven ability to form uniform, homogenous films of well interconnected nanomaterials directly from dilute suspensions.[4, 5] The solution properties of both pristine and carbonized materials relevant to the EPD process were obtained directly from NMP suspensions at a concentration of 0.05 mg/ml active material using a Malvern Zetasizer Nano ZS instrument. Dynamic Light Scattering (DLS) measurements of the zeta potential yielded a zeta potential of -36.8 mV for pristine MoS_2 materials and -42.4 mV for stacked materials. These values are consistent with previously reported values for MoS_2 and carbon nanosheets and serve as further confirmation for the complete passivation of the MoS_2 surface during the stacking process.[6] The average

hydrodynamic diameter for pristine materials in solution (442.8 nm) is over twice that for layered materials (210.1 nm) indicating a higher quality suspension due to the layering process.

Supplementary Note 3. Deconvolution of XPS Data

Deconvolution of the high resolution XPS spectra of the Mo 3d and S 2p binding energies emphasize the influence of strain on the underlying MoS_2 material. Without a carbon interface, the MoS_2 material demonstrate the conventional Mo^{4+} 3d and S 2p components characteristic of 2H-MoS_2. After interfacing with the carbon material, however, additional peaks shifted to lower binding energies emerge that have previously been characterized as the 1T phase in MoS_2 materials.[7] The emergence of this phase in the MoS_2 material is consistent with the notion of interfacial strain as the 2H-1T phase transformation is commonly achieved via the application of applied strain.

Supplementary Note 4. Lithiation Capacity of the Sulfur Conversion Reaction

The discharge curves of both pristine (Figure S3a) and stacked (Figure S3b) materials operated at a constant rate of 100 mA/g are presented below and serve to illustrate the specific capacity of each electrochemical conversion reaction.[8, 9] To evaluate the total capacity of the conversion process, the capacity within a 200 mV window centered about the reaction voltage was considered. In the case of the stacked material, the magnitude of the plateau at ~2.26 V was over 500 mAh/g while that for the pristine sample at ~0.51 V was less than 250 mAh/g indicating a nearly 2x improvement in the efficiency of the vertically stacked MoS_2 conversion reaction compared with the conventional low voltage conversion reaction inherit to MoS_2 material.

The retention of capacity in the converted products upon repeated cycling is depicted in Figure S4a for pristine and stacked materials. A nearly 10% improvement in capacity after 50 cycles is observed for stacked materials while a degradation of over 25% initial capacity is observed for pristine materials. Additional electrochemical impedence spectroscopy (EIS) was performed after each charge-discharge cycle and Nyquist plots for the first 3 cycles are presented in Figure S4b for pristine materials and Figure S4c for vertically stacked materials. The Nyquist plots demonstrate an increase in both the series resistance and charge-transfer resistance for pristine samples upon repeated cycling while carbonized samples demonstrate a decrease in these resistance values. For stacked materials, the presence of a conversion reaction early in the discharge process implies that the large volumetric expansion associated with this reaction faciliates the formation of a higher surface-area solid electrolyte interface (SEI) in the initial discharge process that can help to prevent dissolution of the as-formed lithium polysulfides during subsequent charge-discharge processes.[10]

Supplementary Note 5. Extended cycling of pristine and stacked structures

Repeated cycling of pristine and stacked materials reveals a distinct difference in cycling characteristics. For pristine structures ~70% of the initial capacity is preserved over 50 cycles while stacked architectures demonstrate a 10 % enhancement in capacity. This difference in cycling behavior is attributed to (1) polysulfide dissolution into the electrolyte in pristine MoS_2 that does not occur due to a physical carbon barrier in the C-MoS_2 material and (2) slightly improved connectivity between materials during successive expansion/contraction cycles that "activates" more efficient charge transfer pathways. EIS measurements performed after each discharge reveals the ability of the carbon coating to improve the conductivity of thecomposite electrode in contrast to pristine materials which exhibit a steadily increasing equivalent series resistance (ESR) and charge-transfer resistance.

Supplementary Note 6. MoS_2 Raman intensities during discharge

Raman analysis of the stacked and pristine structures during the initial discharge exhibit a decaying intensity for the MoS_2 E_{2G} and A_{1G} modes as the conversion reaction into polysulfides procedes. At the open circuit condition, comparable intensities are observed between both pristine and stacked materials, however, after discharge to 1.75 V a notable drop in intensity is observed only for stacked structures as the strained MoS_2 material undergoes the high voltage conversion reaction. After a complete discharge to 0.01 V, the characteristic conversion for the pristine MoS_2 material has been completed and we observe comparable intensity for the MoS_2 materials in both cases. The remaining MoS_2 signal intensity is attributed to regions of electrically isolated material in the *ex-situ* cell.

Supplementary Note 7. In-Situ EIS During Initial Discharge

In-situ EIS measurements during the initial discharge for (a) stacked and (b) pristine MoS_2 material enables a direct probe of the interfacial conditions of the material at different states of charge. The corresponding circuits used to provide accurate fits to the data are presented as insets with a table of the fitted values provided in Table S1. At OCV conditions, circuit fitting of EIS measurements of the stacked material demonstrates the presence of two distinct time constants consistent with the presence of capacitive storage at two interfaces within the material, namely, the electrolyte-carbon interface and the carbon-MoS_2 interface. In pristine materials, only one time constant is evident at the OCV conditions and upon discharging to 1.75 V, the interface conditions for the pristine materials remains relatively unchanged while the stacked structures exhibit the formation of single, distinct phase attributed to the formation of a carbon-sulfur composite structure. After discharging to 0.01 V, both materials exhibit comparable interfacial properties, consistent with the notion that a chemically similar composite phase is formed for each material after the initial discharge. Circuit analysis demonstrates comparable values for the resistance of this composite interface in both pristine and stacked material structures as emphasized by Table S1 below.

Supplementary References

1. Acerce, M., Voiry, D. & Chhowalla, M., Metallic 1T phase MoS2 nanosheets as supercapacitor electrode materials. *Nat. Nanotechnol.* **10**, 313-318 (2015).
2. Su, D., Dou, S. & Wang, G., Ultrathin MoS2 Nanosheets as Anode Materials for Sodium-Ion Batteries with Superior Performance. *Adv. Energy Mater.* **5**, 6 (2015).
3. Hui, Y. Y. *et al.*, Exceptional tunability of band energy in a compressively strained trilayer MoS2 sheet. *ACS Nano.* **7**, 7126-7131 (2013).
4. Santhanagopalan, S., Balram, A. & Meng, D. D., Scalable High-Power Redox Capacitors with Aligned Nanoforests of Crystalline MnO2 Nanorods by High Voltage Electrophoretic Deposition. *ACS Nano.* **7**, 2114-2125 (2013).
5. Santhanagopalan, S., Teng, F. & Meng, D. D., High-voltage electrophoretic deposition for vertically aligned forests of one-dimensional nanoparticles. *Langmuir.* **27**, 561-569 (2010).
6. Oakes, L., Hanken, T., Carter, R., Yates, W. & Pint, C. L., Roll-to-Roll Nanomanufacturing of Hybrid Nanostructures for Energy Storage Device Design. *ACS Appl. Mater. Inter.* **7**, 14201-14210 (2015).
7. Eda, G. *et al.*, Photoluminescence from chemically exfoliated MoS2. *Nano Lett.* **11**, 5111-5116 (2011).
8. Bruce, P. G., Freunberger, S. A., Hardwick, L. J. & Tarascon, J.-M., Li-O2 and Li-S batteries with high energy storage. *Nat. Mater.* **11**, 19-29 (2012).
9. Liang, X. *et al.*, A highly efficient polysulfide mediator for lithium–sulfur batteries. *Nat. Commun.* **6**, 5682 (2015).
10. Cao, R., Zhuang, Q.-C., Tian, L.-L., Qiu, X.-Y. & Shi, Y.-L., Electrochemical impedance spectroscopic study of the lithium storage mechanism in commercial molybdenum disulfide. *Ionics* **20**, 459-469 (2014).
